# Enhancing Photocatalytic Activities for Sustainable Hydrogen Evolution on Structurally Matched CuInS_2_/ZnIn_2_S_4_ Heterojunctions

**DOI:** 10.3390/molecules29112447

**Published:** 2024-05-23

**Authors:** Fuying Li, Boiyee Liao, Jinni Shen, Junni Ke, Rongxin Zhang, Yueqi Wang, Yu Niu

**Affiliations:** 1 School of Resources & Chemical Engineering, Sanming University, Sanming 365004, China; afu198207@163.com (F.L.);; 2 Institute of Engineering and Technology Management, Krirk University, Bangkok 10220, Thailand; 3 State Key Laboratory of Photocatalysis on Energy and Environment, Fuzhou University, Fuzhou 350007, China; 4 Fujian Universities Engineering Research Center of Reactive Distillation Technology, Fuzhou University, Fuzhou 350007, China

**Keywords:** CuInS_2_/ZnIn_2_S_4_ photocatalyst, Z-scheme heterojunctions, hydrogen evolution reaction, visible light performance

## Abstract

Effective charge separation and migration pose a critical challenge in the field of solar-driven hydrogen production. In this work, a Z-scheme structured CuInS_2_/ZnIn_2_S_4_ heterojunction was successfully fabricated through a two-step hydrothermal synthesis method to significantly enhance the efficiency of solar-to-hydrogen energy conversion. Structural characterization revealed that the lattice-matched CuInS_2_/ZnIn_2_S_4_ heterojunction exhibits an enlarged interfacial contact area, which facilitates the transfer and separation of photogenerated charges. Microscopic analysis indicated that the CuInS_2_/ZnIn_2_S_4_ composite material has a tightly interwoven interface and a morphology resembling small sugar cubes. Photoelectrochemical spectroscopy analysis demonstrated that the heterojunction structure effectively enhances visible light absorption and charge separation efficiency, leading to an improvement in photocatalytic activity. Hydrogen production experimental data indicated that the CuInS_2_/ZnIn_2_S_4_ heterojunction photocatalyst prepared with a CuInS_2_ content of 20 wt% exhibits the highest hydrogen evolution rate, reaching 284.9 μmol·g^−1^·h^−1^. Moreover, this photocatalyst maintains robust photocatalytic stability even after three consecutive usage cycles. This study demonstrated that the Z-scheme CuInS_2_/ZnIn_2_S_4_ heterojunction photocatalyst exhibits enhanced hydrogen evolution efficiency, offering an effective structural design for harnessing solar energy to obtain hydrogen fuel. Therefore, this heterojunction photocatalyst is a promising candidate for practical applications in solar hydrogen production.

## 1. Introduction

Transforming solar energy into a sustainable and clean energy source is a vital approach for addressing the current challenges of energy scarcity and environmental pollution [1,2,3]. The utilization of semiconductor photocatalysts for the decomposition of water to produce hydrogen represents a promising technology for addressing the energy crisis [4,5,6,7]. However, the development of highly efficient photocatalysts will be pivotal for improving the feasibility of industrializing this technology [8,9]. Potential semiconductor photocatalysts should minimize the rapid recombination of photogenerated charge carriers, and they should be responsive to both ultraviolet and visible light [10,11]. Photocatalysts such as ZnIn_2_S_4_ have been extensively investigated due to their non-toxicity and suitable bandgap widths, which render them effective for the photocatalytic splitting of water to generate hydrogen. However, the photocatalytic water-splitting performance of pure ZnIn_2_S_4_ for hydrogen production is compromised by the severe recombination of photogenerated charges. Researchers have employed strategies such as doping or coupling with other semiconductor photocatalysts to overcome these deficiencies [12,13]. Excitingly, the formation of heterojunction structures within photocatalysts can significantly enhance the efficiency of photogenerated charge separation [14].

Therefore, in recent years, researchers have studied new heterojunction photocatalysts. Lou et al. reported that the formation of ZnIn_2_S_4_-In_2_O_3_ heterojunctions [15] improved the separation and transfer of photogenerated charges. Chen et al. reported that the photocatalytic H_2_ production rate of a ZrC@ZnIn_2_S_4_ core–shell heterostructure reached 32.87 μmol·g^−1^·h^−1^, and the formation of a Schottky junction accelerated the photogenerated transfer and separation of charge carriers [16]. Xie et al. reported that a direct Z-scheme ZnIn_2_S_4_/ZrO_2_ heterojunction improved photocatalytic properties, which was ascribed to the heterojunction accelerating photo-induced carrier separation [17]. Pu et al. reported a ZnIn_2_S_4_/Cu_2_MoS_4_ S-scheme heterostructure that efficiently facilitated the separation and transfer of light-induced charges [18]. Li et al. reported a ZnIn_2_S_4_/CdS hollow core–shell nano-heterostructure with an appropriate potential gradient and Zn/In bimetallic synergism, showing improved carrier transportation properties [19]. Research has revealed that constructing heterojunctions between two or more semiconductors results in the creation of a potential gradient and an internal electric field, which serves as an effective method for the separation of electrons and holes [20,21]. However, previous studies have mainly considered semiconductor bandgap width matching, whose perspective of structural matching has not been investigated as thoroughly. Evaluating the morphological lattice match between photocatalyst components will be essential for charge separation and transfer at the interface [22].

CuInS_2_ is a promising semiconductor photocatalyst because it possesses the ability to absorb visible light, which is a predominant component of solar radiation. Thus, CuInS_2_ has shown potential for application across a range of fields, including the photocatalytic splitting of water for hydrogen production, the photocatalytic reduction of CO_2_, and the degradation of organic pollutants under photocatalytic conditions [23,24,25,26,27]. ZnIn_2_S_4_ and CuInS_2_ are both ternary sulfides that have similar morphologies and structures, and they exhibit a high degree of lattice matching. The in situ growth of CuInS_2_ on ZnIn_2_S_4_ can yield a Z-scheme heterojunction with tightly interconnected interfaces, which is conducive to enhancing the interfacial contact area, accelerating the charge migration rate, and suppressing the recombination of electron–hole pairs [28]. Therefore, the synergistic effect of CuInS_2_/ZnIn_2_S_4_ Z-scheme heterojunctions can be expected to lead to highly efficient photocatalytic H_2_ production.

In this study, a facile solvothermal method was employed to grow CuInS_2_ on the surface of ZnIn_2_S_4_, resulting in the synthesis of CuInS_2_/ZnIn_2_S_4_ Z-scheme heterojunction nano-photocatalysts with a small-sugar-cube-like morphology. The hydrogen evolution performance of this composite material is significantly superior to that of pure ZnIn_2_S_4_. By characterizing the structural, electrochemical, and spectral properties of this catalyst, the reasons behind the enhanced photocatalytic activity of CuInS_2_/ZnIn_2_S_4_ were analyzed.

Photocatalytic activity for hydrogen production via water splitting under visible light was evaluated using ethylene glycol as a sacrificial agent. The results indicated that the incorporation of an appropriate amount of CuInS_2_ enhances visible light absorption, suppresses the recombination of photogenerated charges, and accelerates the migration of carriers. Therefore, photocatalytic hydrogen evolution performance and stability are improved. The possible mechanisms underlying the enhancement of photocatalysis were discussed. This research offers a potential pathway for the rational design and construction of Z-scheme heterostructure photocatalysts, which facilitate the separation and migration of photogenerated charge carriers and enhance the performance of hydrogen production via water splitting under visible light.

## 2. Results and Discussion

### 2.1. X-ray Diffraction (XRD)

**Figure 1:**
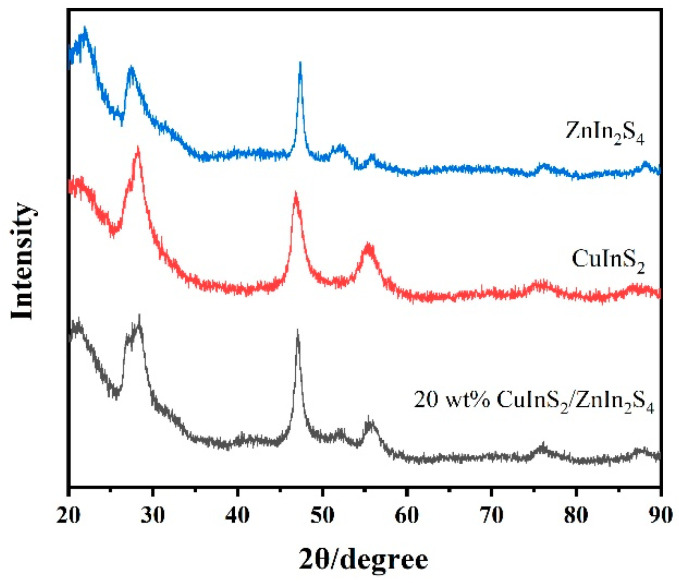
XRD patterns of CuInS_2_, ZnIn_2_S_4_, and CuInS_2_/ZnIn_2_S_4_ photocatalysts.

XRD analysis (Figure 1) indicates that the pure CuInS_2_ and ZnIn_2_S_4_ photocatalysts have high crystallinity [29], as indicated by the absence of impurity diffraction peaks. The XRD pattern of CuInS_2_ exhibits strong diffraction peaks at 28.3°, 46.7°, and 55.1°, which, respectively, correspond to the (112), (204), and (116) lattice planes of tetragonal CuInS_2_. These diffraction peaks match well with the standard card of CuInS_2_ (JCPDS: 32-0339). Similarly, the XRD pattern of ZnIn_2_S_4_ exhibits strong diffraction peaks at 26.9°, 47.4°, 51.8°, and 55.9°, which, respectively, correspond to the (102), (110), (116), and (202) lattice planes of hexagonal ZnIn_2_S_4_. These peaks correspond to the standard card of ZnIn_2_S_4_ (JCPDS: 65-2023). The XRD results indicate that the CuInS_2_, ZnIn_2_S_4_, and composite CuInS_2_/ZnIn_2_S_4_ photocatalysts were successfully prepared by the two-step hydrothermal method. The sample doped with 20 wt% CuInS_2_ exhibits diffraction peaks ascribed to both ZnIn_2_S_4_ and CuInS_2_, suggesting that the crystal structures of both ZnIn_2_S_4_ and CuInS_2_ were preserved during the synthesis process without being compromised. This unique structural configuration plays a critical role in enhancing the photocatalytic performance of CuInS_2_/ZnIn_2_S_4_.

### 2.2. Scanning Electron Microscopy (SEM)

**Figure 2:**
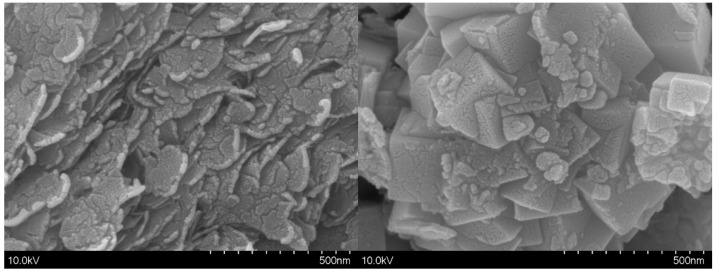
SEM images of pure ZnIn_2_S_4_ and 20 wt% CuInS_2_/ZnIn_2_S_4_.

The morphologies of pure ZnIn_2_S_4_ and 20 wt% CuInS_2_/ZnIn_2_S_4_ were investigated using SEM. As shown in Figure 2, pure ZnIn_2_S_4_ exhibits an overlapping flaky morphology composed of numerous thin nanosheets with thicknesses in the range of 10–20 nm. The surface of pure ZnIn_2_S_4_ is rough and uneven, showing noticeable signs of fragmentation. In contrast, the 20 wt% CuInS_2_/ZnIn_2_S_4_ composite sample morphology consists of nanocubes with sizes of around 100–200 nm. Thus, 20 wt% CuInS_2_/ZnIn_2_S_4_ has a uniformly dispersed sugar-cube-like appearance. Furthermore, CuInS_2_ nanoparticles with sizes of about 10–50 nm are evenly distributed across the surface of the ZnIn_2_S_4_ nanocubes, indicating the formation of a Z-scheme heterojunction between CuInS_2_ and ZnIn_2_S_4_ [30]. These experimental results demonstrate that during the in situ hydrothermal synthesis of the CuInS_2_/ZnIn_2_S_4_ composites, controlling the degree of crystallization plays a role in regulating the growth and dispersion of the CuInS_2_ nanoparticles on the surface of the ZnIn_2_S_4_ nanocubes. This is expected to significantly impact photocatalytic performance. However, the precise mechanism by which CuInS_2_ nanoparticles grow on the surface of ZnIn_2_S_4_ still requires further investigation. Controlling the synthesis reaction conditions may be an effective strategy for optimizing the size distribution and dispersion of the nanoparticles, which is essential for the development of nano-heterojunction composite photocatalysts with enhanced photocatalytic performance.

### 2.3. UV–Vis Diffuse Reflection Spectroscopy (DRS)

**Figure 3:**
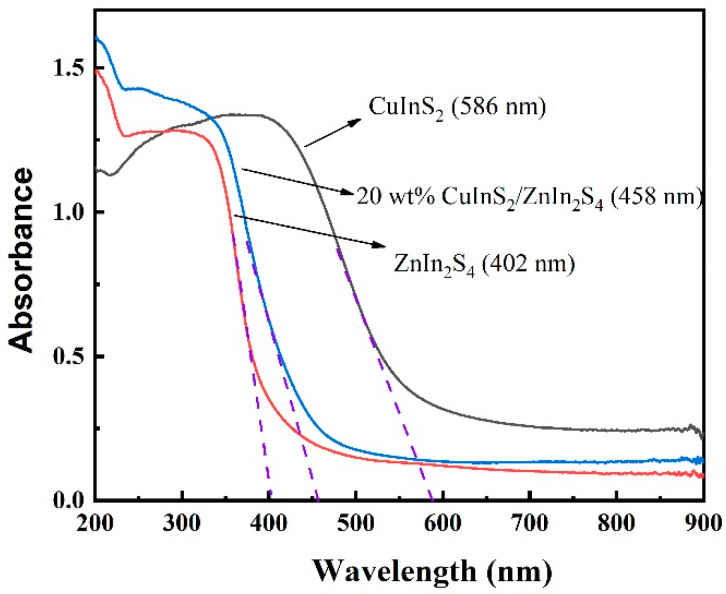
UV–Vis DRS spectra of pure ZnIn_2_S_4_, pure CuInS_2_, and 20 wt% CuInS_2_/ZnIn_2_S_4_.

DRS spectra of pure ZnIn_2_S_4_, pure CuInS_2_, and 20 wt% CuInS_2_/ZnIn_2_S_4_ were obtained to investigate the impact of CuInS_2_ on the light absorption properties of ZnIn_2_S_4_, as displayed in Figure 3. The results indicate that all samples exhibit strong absorption in the visible light region. However, pure ZnIn_2_S_4_ can only absorb light with wavelengths shorter than 402 nm. In contrast, the optical absorption edge of CuInS_2_ extends to 586 nm, indicating that CuInS_2_ has a superior visible light response compared to ZnIn_2_S_4_. The 20 wt% CuInS_2_/ZnIn_2_S_4_ composite material exhibits a significant redshift at a wavelength of 458 nm, which suggests that the prepared composite sample absorbs visible light more strongly compared to pure ZnIn_2_S_4_. Therefore, the introduction of CuInS_2_ significantly enhances the absorption efficiency of visible light, which is beneficial for generating a greater number of photogenerated electrons and holes. Moreover, the improved visible light absorption performance of the CuInS_2_/ZnIn_2_S_4_ composite also indicates the successful construction of the Z-scheme heterojunction. 

**Figure 4:**
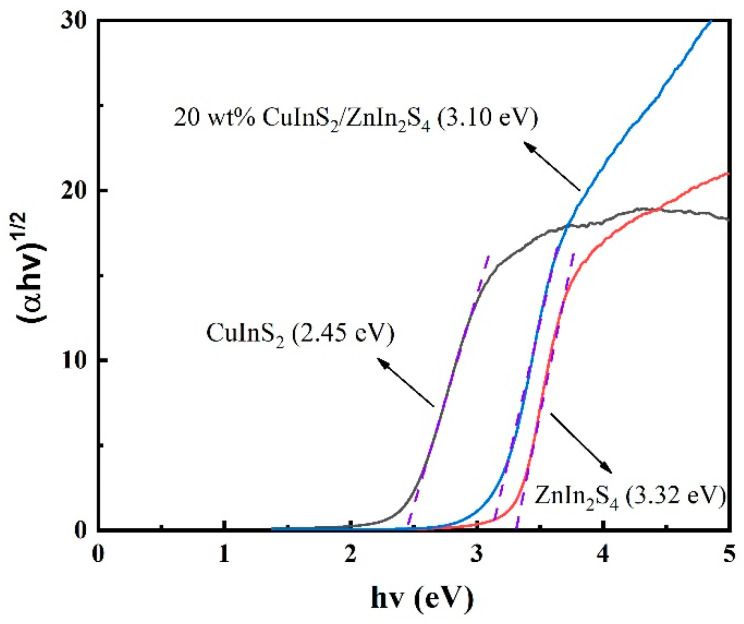
Transformed Kubelka–Munk function versus photon energy plots of ZnIn_2_S_4_, CuInS_2_, and 20 wt% CuInS_2_/ZnIn_2_S_4_.

Plots of (ahv)^1/2^ versus (hv) were obtained using the Kubelka–Munk function, as shown in Figure 4. The bandgap energy of pure ZnIn_2_S_4_ is approximately 3.32 eV, while pure CuInS_2_ has a narrower bandgap energy of about 2.45 eV, demonstrating its stronger ability to absorb visible light. The bandgap energy of the 20% wt% CuInS_2_/ZnIn_2_S_4_ composite material is 3.10 eV, which falls between that of pure ZnIn_2_S_4_ and pure CuInS_2_. This may be attributed to the formation of a heterojunction and new photogenerated electron transfer pathways at the interface between the two semiconductors. Consequently, compared to pure ZnIn_2_S_4_, 20 wt% CuInS_2_/ZnIn_2_S_4_ can absorb and utilize visible light more effectively, thereby enhancing the efficiency of photocatalytic hydrogen production via water splitting.

### 2.4. Transient Photocurrent Response (TPR) and Electrochemical Impedance Spectroscopy (EIS)

**Figure 5:**
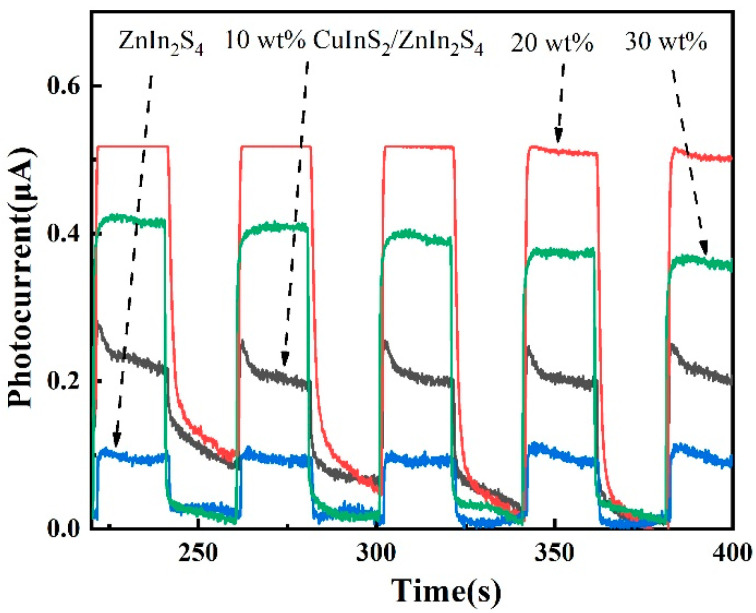
TPR curves of ZnIn_2_S_4_, CuInS_2_, and CuInS_2_/ZnIn_2_S_4_ samples under intermittent visible light irradiation.

To investigate the superior photoresponse and charge separation transfer characteristics of the Z-scheme structure in the CuInS_2_/ZnIn_2_S_4_ composite materials, the TPRs of the photocatalysts were measured under visible light illumination, as presented in Figure 5. The CuInS_2_/ZnIn_2_S_4_ composite samples exhibit higher photocurrent densities compared to pure ZnIn_2_S_4_. With increasing CuInS_2_ content, the photocurrent density initially increases and then decreases. Among the composite photocatalysts, the sample with 20 wt% CuInS_2_ demonstrates the highest photocurrent density, suggesting that this heterojunction photocatalyst generates a greater number of photoexcited electrons under visible light illumination per unit time.

The TPR curve of the 10 wt% CuInS_2_/ZnIn_2_S_4_ sample shows a sharp peak in the photocurrent density value at the initial stage of irradiation, which can be attributed to the recombination of photogenerated carriers. In other words, the photogenerated electrons in ZnIn_2_S_4_ cannot quickly migrate to the surface and are captured by photogenerated holes, leading to a decay in photocurrent density. However, this phenomenon is not observed in the curves of the 20 wt% and 30 wt% CuInS_2_/ZnIn_2_S_4_ samples. Therefore, the recombination of photogenerated electrons and holes is inhibited in 20 wt% and 30 wt% CuInS_2_/ZnIn_2_S_4_, which allows these samples to maintain stable photocurrent densities throughout the photoresponse process. This suggests that CuInS_2_ and ZnIn_2_S_4_ have matching lattice structures and that the formation of a Z-scheme heterojunction can effectively migrate photogenerated carriers, reducing the recombination degree of photogenerated electrons and holes. Consequently, the photocurrent density of the 20 wt% CuInS_2_/ZnIn_2_S_4_ composite sample is more than five times higher than that of pure ZnIn_2_S_4_. This significant enhancement in photocurrent density highlights the effectiveness of the composite CuInS_2_/ZnIn_2_S_4_ material in promoting the separation and migration of photogenerated charge carriers, which plays a crucial role in improving the efficiency of photocatalytic water splitting for hydrogen production.

**Figure 6:**
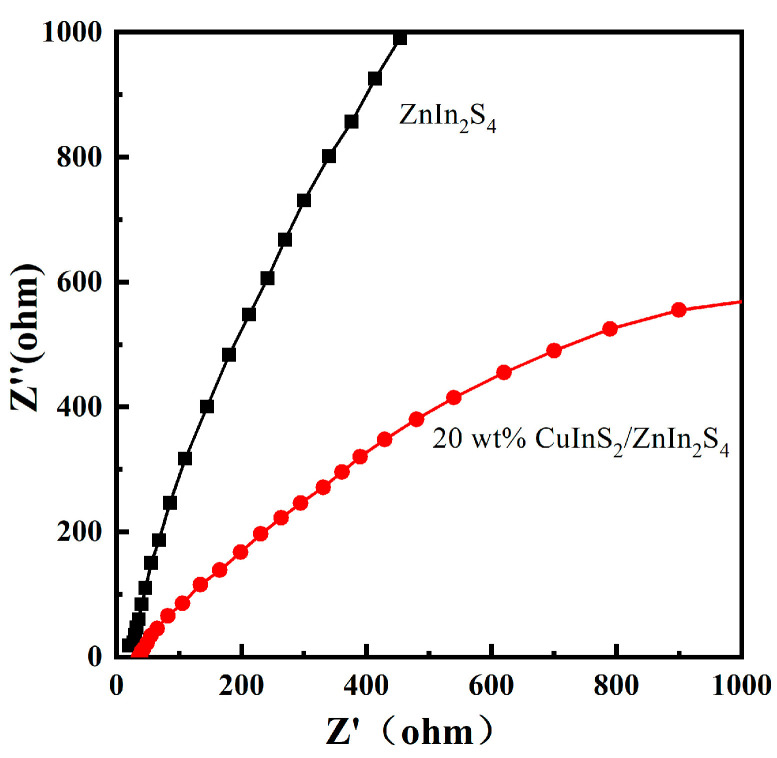
EIS curves of pure ZnIn_2_S_4_ and 20 wt% CuInS_2_/ZnIn_2_S_4_.

The EIS curves of pure ZnIn_2_S_4_ and the 20 wt% CuInS_2_/ZnIn_2_S_4_ composite material are displayed in Figure 6. A smaller semicircle diameter indicates less resistance to the migration of photogenerated charges within the catalyst structure. The semicircle diameter of the 20 wt% CuInS_2_/ZnIn_2_S_4_ composite sample is noticeably smaller than that of pure ZnIn_2_S_4_, suggesting that this composite photocatalyst structure has less resistance to charge migration, which is beneficial for faster charge separation and transport. The easier migration of photogenerated charges to the catalyst surface to participate in redox reactions enhances the photocatalytic performance for hydrogen production from water splitting.

### 2.5. Photocatalytic Performance for Hydrogen Evolution Reaction (HER)

**Figure 7:**
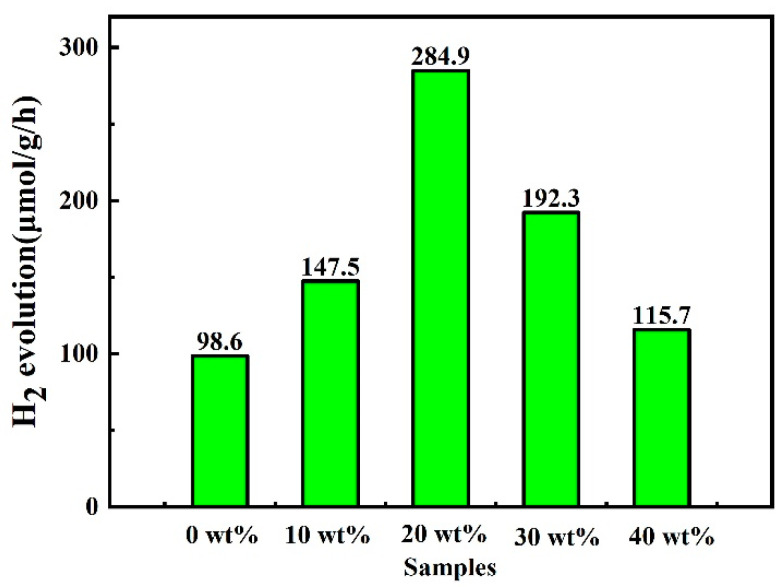
Photocatalytic HER rates of pure ZnIn_2_S_4_ and CuInS_2_/ZnIn_2_S_4_ composites.

Hydrogen production experiments were conducted under visible light (λ ≥ 400 nm) using triethanolamine (TEOA) as a sacrificial agent in deionized water. Photocatalytic activity for the HER was evaluated using online analytical testing equipment to assess the impact of varying the CuInS_2_ content on the photocatalytic HER activity of the CuInS_2_/ZnIn_2_S_4_ composite photocatalysts. Pure CuInS_2_ produces almost no hydrogen due to its low photocatalytic activity. The CuInS_2_/ZnIn_2_S_4_ composite photocatalysts exhibit higher photocatalytic hydrogen evolution rates compared to pure ZnIn_2_S_4_, indicating that the Z-scheme heterojunction structure is an effective design for enhancing the efficiency of photocatalytic hydrogen production. Figure 7 presents the hydrogen production rate curves of the heterojunction photocatalysts prepared with different mass fractions of CuInS_2_. Due to severe charge recombination, ZnIn_2_S_4_ exhibits a hydrogen production rate of 98.6 μmol·g^−1^·h^−1^, indicating relatively low photocatalytic activity. However, the samples loaded with CuInS_2_ show significantly enhanced photocatalytic hydrogen evolution activity. Notably, 20 wt% CuInS_2_/ZnIn_2_S_4_ demonstrates the highest photocatalytic hydrogen evolution rate of 284.9 μmol·g^−1^·h^−1^, which is three times that of pure ZnIn_2_S_4_. The hydrogen production rates of the 10 wt%, 30 wt%, and 40 wt% CuInS_2_/ZnIn_2_S_4_ photocatalysts are 147.5 μmol·g^−1^·h^−1^, 192.3 μmol·g^−1^·h^−1^, and μmol·g^−1^·h^−1^, respectively. The CuInS_2_ content of these heterojunction samples significantly influences their photocatalytic activity. The underlying photocatalytic mechanism is attributed to the dynamics of photogenerated charge transfer and carrier utilization efficiency [31], which are crucial for achieving enhanced photocatalytic efficiency.

**Figure 8:**
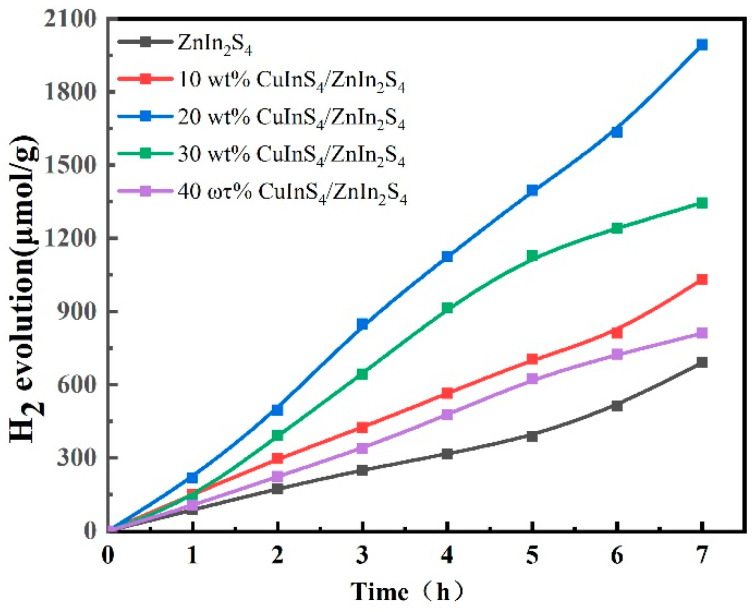
Time-dependent photocatalytic HER performance of pure ZnIn_2_S_4_ and CuInS_2_/ZnIn_2_S_4_ composites.

Figure 8 illustrates the relationship between hydrogen production and time for pure ZnIn_2_S_4_ and the CuInS_2_/ZnIn_2_S_4_ composite materials with varying CuInS_2_ content. The photocatalytic hydrogen production yields of these samples increase linearly with time. Among the CuInS_2_/ZnIn_2_S_4_ heterojunction photocatalysts, the catalyst prepared with 20% wt% CuInS_2_ exhibits the highest hydrogen production rate, with a yield of approximately 1994 μmol·g^−1^ after 7 h. This yield is three times that of pure ZnIn_2_S_4_. These results suggest that the optimal CuInS_2_ loading amount for preparing the heterojunction composite photocatalyst is 20%.

**Figure 9:**
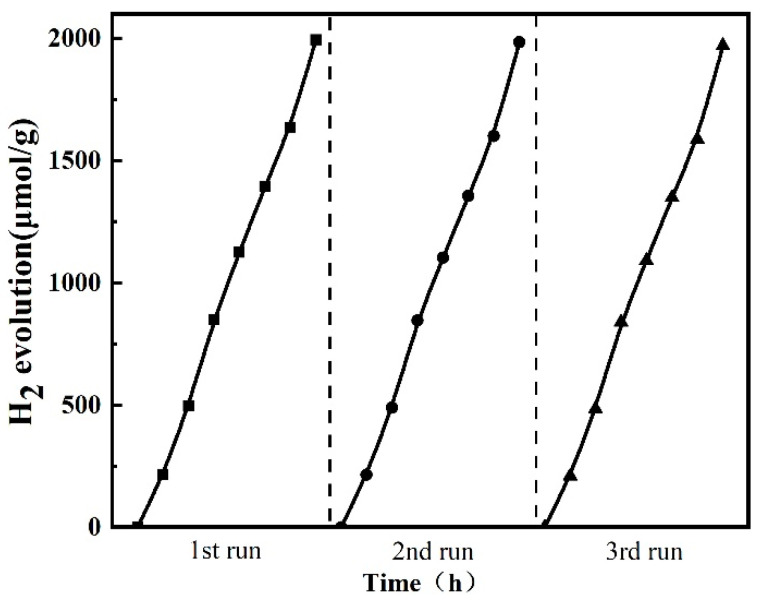
Photocatalytic stability of 20 wt% CuInS_2_/ZnIn_2_S_4_ for photocatalytic HER.

The stability of a photocatalyst is a critical factor affecting its practical application. Therefore, the reusability and stability of the 20 wt% CuInS_2_/ZnIn_2_S_4_ photocatalyst were assessed by collecting and reusing the same catalyst in three cycles under the same experimental conditions, as shown in Figure 9. The results indicate that the 20 wt% CuInS_2_/ZnIn_2_S_4_ sample does not show a significant decline in photocatalytic activity, and H_2_ production remains stable after three cycles. This can be attributed to the effective transfer of photogenerated electrons from CuInS_2_ to ZnIn_2_S_4_ in the CuInS_2_/ZnIn_2_S_4_ nano-heterojunction, which inhibits the photocorrosion effect caused by photogenerated holes.

### 2.6. Photocatalytic HER Mechanism

**Figure 10:**
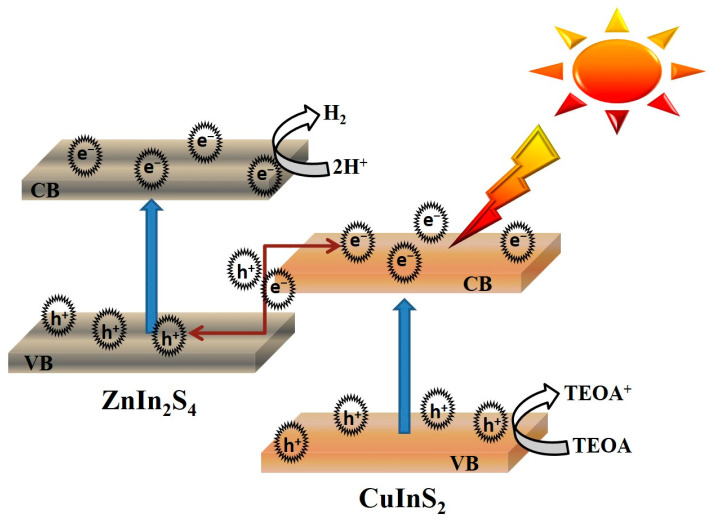
Schematic diagram of charge separation and transfer process within the Z-scheme CuInS_2_/ZnIn_2_S_4_ heterojunction photocatalyst.

The mechanism of photocatalytic water splitting for hydrogen production under simulated solar light in the Z-scheme CuInS_2_/ZnIn_2_S_4_ heterojunction photocatalyst is presented in Figure 10. This hydrogen evolution mechanism involves the transfer of photogenerated electrons from the conduction band (CB) of CuInS_2_ to the valence band (VB) of ZnIn_2_S_4_. The photogenerated electrons produced by ZnIn_2_S_4_ can then participate in the HER. The migration mechanism of photogenerated charge carriers within the heterojunction is attributed to the differing band positions of CuInS_2_ and ZnIn_2_S_4_ [32]. The CB of ZnIn_2_S_4_ is positioned higher than that of CuInS_2_, allowing electrons in the CB of CuInS_2_ to transfer to the VB of ZnIn_2_S_4_, where they can participate in the electron–hole recombination process. Conversely, the VB of ZnIn_2_S_4_ is lower than that of CuInS_2_, enabling holes in the VB of ZnIn_2_S_4_ to transfer to the more positive VB of CuInS_2_. Moreover, the holes that accumulate in the VBs of CuInS_2_ and ZnIn_2_S_4_ can be rapidly consumed by the sacrificial reagent. This process reduces photocorrosion damage and enhances the structural stability of the composite photocatalyst, thereby extending its service life and improving its photocatalytic performance. Furthermore, the synergistic effect of the CuInS_2_/ZnIn_2_S_4_ composite material originates from its well-matched band structure and the formation of a Z-scheme heterojunction, which is facilitated by their similar composition and structure [33,34]. The closely connected interface between CuInS_2_ and ZnIn_2_S_4_ provides a reliable and stable pathway for charge transfer, thereby enhancing charge separation [35,36] and broadening light absorption within the visible light spectrum. This improves photocatalytic activity and contributes to the stability of the catalyst. Charge transfer leads to the formation of an internal electric field at the Z-scheme heterojunction interface, which facilitates the separation and migration of charge carriers, thereby enhancing the overall photocatalytic performance [37,38]. In summary, the unique mechanism of the CuInS_2_/ZnIn_2_S_4_ composite photocatalyst offers a promising pathway for efficient hydrogen production. The effective separation and utilization of photogenerated charge carriers coupled with the rapid consumption of holes by a sacrificial agent demonstrate the strong potential of CuInS_2_/ZnIn_2_S_4_ as a candidate for applications in solar-driven hydrogen production.

In summary, the in situ two-step hydrothermal method employed herein represents an effective strategy for preparing heterostructure photocatalysts. By controlling the crystal morphology during the photocatalyst synthesis process, this method leads to the formation of highly stable and robust structures. The obtained CuInS_2_/ZnIn_2_S_4_ composite material is a promising photocatalyst for HER.

## 3. Experimental Section

### 3.1. Photocatalyst Preparation

All chemicals were of reagent grade and were used as received without any further purification.

#### 3.1.1. Preparation of CuInS_2_ and ZnIn_2_S_4_

CuInS_2_ and ZnIn_2_S_4_ photocatalysts were synthesized using a one-step hydrothermal method. To prepare ZnIn_2_S_4_, C_4_H_6_O_4_Zn·2H_2_O (1.0 mmol), In(NO_3_)_3_·4H_2_O (2.0 mmol), and excess C_2_H_5_SN (8.0 mmol) were added to 60 mL of deionized water. This solution was stirred and sonicated for 0.5 h to achieve thorough mixing. Next, the mixture was transferred to a Teflon-lined autoclave and heated at 160 °C for 18 h. The obtained powder was washed with deionized water and ethanol three times. The washed solid was dried at 60 °C in air for 12 h and ground for 1 h. The resulting product was designated as ZnIn_2_S_4_. CuInS_2_ was obtained using the same process.

#### 3.1.2. Preparation of CuInS_2_/ZnIn_2_S_4_

The CuInS_2_/ZnIn_2_S_4_ composites were synthesized using a two-step hydrothermal method. A mixture of CuCl, In(NO_3_)_3_·4H_2_O, and C_2_H_5_SN was dissolved in 40 mL of deionized water. Next, a certain amount of ZnIn_2_S_4_ powder (to achieve a CuInS_2_ and ZnIn_2_S_4_ mass ratio of 10%, 20%, 30%, or 40%) was added to this solution. The resulting mixture was stirred and sonicated for 0.5 h to achieve thorough mixing. The solution was then transferred to a Teflon-lined autoclave and heated at 160 °C for 18 h. Each obtained powder was washed with deionized water and ethanol three times, then dried at 60 °C in air for 12 h and ground for 1 h. The products were labeled as 10 wt% CuInS_2_/ZnIn_2_S_4_, 20 wt% CuInS_2_/ZnIn_2_S_4_, 30 wt% CuInS_2_/ZnIn_2_S_4_, and 40 wt% CuInS_2_/ZnIn_2_S_4_.

### 3.2. Characterization of Photocatalysts

XRD was used to analyze the phase compositions of the samples. SEM was used to study the microstructures of the samples. UV–Vis spectroscopy was used to record the DRS of the samples. An electrochemical workstation was used to determine the TPR and EIS of the samples. A platinum foil served as the counter electrode, and a saturated calomel electrode (SCE) was used as the reference electrode. The working electrode was prepared by coating a 0.5 × 0.5 cm^2^ area of a fluorine-doped tin oxide (FTO) glass with each prepared photocatalyst sample. This three-electrode system was fully immersed in a 0.5 M Na_2_SO_4_ electrolyte solution. The working electrode was subjected to irradiation from a light source with a wavelength of 365 nm.

### 3.3. Photocatalytic HER Tests

The photocatalytic HER tests were conducted in a 250 mL quartz tube reactor, which was part of an online photocatalytic test system (Labsolar-6A, Beijing Perfect Light, Beijing, China). A 300 W Xe lamp (λ ≥ 400 nm) was used as the light source to provide simulated AM 1.5 irradiation (100 mW·cm^−2^, as measured by a PL-MW2000 Optical power meter, Beijing Perfect Light, Beijing, China). In a typical experiment, 25 mg of solid catalyst powder was dispersed using sonication in a solution containing 90 mL of deionized water and 10 mL of TEOA. The reactor was evacuated and purged with high-purity nitrogen prior to the photocatalytic reaction. The reaction was carried out at room temperature for 7 h. The hydrogen content was analyzed using an Micro GC3000 (Agilent, Santa Clara, CA, USA) equipped with a 5A molecular sieve column and a high-sensitivity online thermal conductivity detector.

## 4. Conclusions

In this study, a two-step hydrothermal method was employed to synthesize CuInS_2_/ZnIn_2_S_4_ composite photocatalysts. Various characterization techniques, including XRD, SEM, UV–Vis spectroscopy, and photoelectrochemical measurements, were utilized to investigate the structure, morphology, light absorption, and impedance of the prepared samples. XRD analysis confirmed the successful synthesis of the ternary chalcogenide CuInS_2_/ZnIn_2_S_4_ composite material. SEM analysis revealed that the 20 wt% CuInS_2_/ZnIn_2_S_4_ sample exhibits a uniformly dispersed sugar-cube-like morphology, demonstrating the formation of a Z-scheme heterojunction. The CuInS_2_ particles, whose diameters range from 10 to 50 nm, are evenly distributed across the surface of the ZnIn_2_S_4_ nanocubes. Thus, the CuInS_2_ nanoparticles are well integrated with the ZnIn_2_S_4_ nanoblocks. The incorporation of CuInS_2_ significantly broadens the wavelength range of visible light absorption by the composite materials compared to pure ZnIn_2_S_4_. The adsorption edge of the 20 wt% CuInS_2_/ZnIn_2_S_4_ composite is redshifted to 458 nm, indicating its enhanced ability to utilize visible light. This redshift is attributed to the formation of a Z-scheme heterojunction between CuInS_2_ and ZnIn_2_S_4_, which facilitates the generation of more photoexcited electron–hole pairs and notably improves the separation efficiency of photogenerated charge carriers. The transient photocurrent density of the 20 wt% CuInS_2_/ZnIn_2_S_4_ composite is five times greater than that of pure ZnIn_2_S_4_. This increase in photocurrent density is indicative of higher light utilization efficiency for the photocatalytic process. Furthermore, the 20 wt% CuInS_2_/ZnIn_2_S_4_ composite has a reduced Nyquist plot semicircle diameter, suggesting lower internal resistance to charge transfer. This decrease in resistance accelerates the separation and transportation of photogenerated charges, which is a critical factor in enhancing the overall photocatalytic performance.

Comparative catalytic activity experiments demonstrate that the 20 wt% CuInS_2_/ZnIn_2_S_4_ composite exhibits superior photocatalytic activity for hydrogen production through water splitting. The hydrogen production rate of this photocatalyst reaches 284.9 μmol·g^−1^·h^−1^, which is three times that of pure ZnIn_2_S_4_ under the same conditions. This indicates a significant enhancement in the efficiency of photocatalytic hydrogen evolution due to the incorporation of CuInS_2_ into the ZnIn_2_S_4_ matrix. The linear relationship between the amount of hydrogen produced and the reaction time further confirms the consistent and sustained photocatalytic performance of the 20 wt% CuInS_2_/ZnIn_2_S_4_ composite. Therefore, this composite material is capable of effectively utilizing light energy to drive the water-splitting process over an extended period, which is a desirable characteristic for practical applications. A catalyst stability experiment shows that the 20 wt% CuInS_2_/ZnIn_2_S_4_ composite material can maintain a consistent hydrogen production rate across three consecutive cycles of use with no significant degradation in catalytic activity. The CuInS_2_/ZnIn_2_S_4_ Z-scheme heterojunction demonstrates exceptional photocatalytic performance and stability, making it a promising material for applications in solar-driven water splitting for hydrogen production and the utilization of clean energy.

## Data Availability

Data are contained within the article.
